# Conformational Selection and Submillisecond Dynamics of the Ligand-binding Domain of the *N*-Methyl-d-aspartate Receptor*

**DOI:** 10.1074/jbc.M116.721274

**Published:** 2016-05-21

**Authors:** Drew M. Dolino, Soheila Rezaei Adariani, Sana A. Shaikh, Vasanthi Jayaraman, Hugo Sanabria

**Affiliations:** From the ‡ Department of Biochemistry and Molecular Biology, Center for Membrane Biology, University of Texas Health Science Center, Graduate School for Biomedical Sciences, Houston, Texas 77030 and; the § Department of Physics and Astronomy, Clemson University, Clemson, South Carolina 29634

**Keywords:** biophysics, fluorescence resonance energy transfer (FRET), glutamate receptor, ion channel, ionotropic glutamate receptor, N-methyl-d-aspartate receptor (NMDA receptor, NMDAR), protein conformation, single-molecule biophysics

## Abstract

The *N*-methyl-d-aspartate (NMDA) receptors are heteromeric non-selective cation channels that require the binding of glycine and glutamate for gating. Based on crystal structures, the mechanism of partial agonism at the glycine-binding site is thought to be mediated by a shift in the conformational equilibrium between an open clamshell and a closed clamshell-like structure of the bilobed ligand-binding domain (LBD). Using single-molecule Förster resonance energy transfer (smFRET) and multiparameter fluorescence detection, which allows us to study the conformational states and dynamics in the submillisecond time scale, we show that there are at least three conformational states explored by the LBD: the low FRET, medium FRET, and high FRET states. The distance of the medium and low FRET states corresponds to what has been observed in crystallography structures. We show that the high FRET state, which would represent a more closed clamshell conformation than that observed in the crystal structure, is most likely the state initiating activation, as evidenced by the fact that the fraction of the protein in this state correlates well with the extent of activation. Furthermore, full agonist bound LBDs show faster dynamic motions between the medium and high FRET states, whereas they show slower dynamics when bound to weaker agonists or to antagonists.

## Introduction

Ionotropic glutamate receptors are a family of ligand-gated ion channels that include the α-amino-3-hydroxy-5-methyl-4-isoxazolepropionic acid receptor, the kainate receptor, and the *N*-methyl-d-aspartate (NMDA) receptor (1). The neurotransmitter glutamate binds to the ionotropic glutamate receptors at the aptly named ligand-binding domain (LBD),^4^ an extracellular domain of the protein that is organized in a clamshell-shaped fold. The LBD, in the resting conformation, has an open cleft, and the binding of glutamate or other agonists induces a closure of the clamshell cleft. This initial conformational change induces a series of other changes (2), leading to the opening of the ion channel, the passing of cations across the postsynaptic membrane, and the propagation of the electrical signal to the postsynaptic neuron.

Because of this pivotal role of the LBD to the ionotropic glutamate receptors, numerous groups have examined the link between LBD conformation and ionotropic glutamate receptor function. Various studies into the α-amino-3-hydroxy-5-methyl-4-isoxazolepropionic acid receptor LBD have revealed a graded cleft closure mechanism, whereas full agonists such as glutamate, which fully activate the channel, also fully close the clamshell cleft; partial agonists, which only partially activate the channel, seem to stabilize a partially closed conformational intermediate (3–6). Thus, activation of the α-amino-3-hydroxy-5-methyl-4-isoxazolepropionic acid receptor appears to be dictated by the extent to which an agonist can close its LBD cleft (7, 8). Ensemble-based dynamic studies using a luminescence resonance energy transfer technique also show a similar mechanism with the glutamate-binding LBD of the NMDA receptor (9).

In comparison to the other ionotropic glutamate receptors, the NMDA receptors are unique in that they are obligate heteromers typically consisting of a glutamate-binding subunit such as GluN2A and the glycine-binding subunit GluN1. As before, when its ligand binds to the GluN1 subunit LBD, it closes its clamshell cleft. However, crystal studies of the GluN1 LBD show no difference in the extent of cleft closure with different partial agonists, leading to the hypothesis of a two-state model, in which the closed, active state is stabilized to varying degrees, rather than the multistate model of the α-amino-3-hydroxy-5-methyl-4-isoxazolepropionic acid receptor (10–13). Consistent with this, computational experiments using umbrella sampling methods have revealed that the apo-GluN1 LBD conformational landscape shows two free energy minima, with one minimum corresponding to a closed clamshell and the other to an open clamshell (14, 15). Together, this suggests that the addition of agonist stabilizes the closed clamshell conformation with partial agonists stabilizing the conformation to a lesser degree.

Experimental verification of the two-state model has been attempted using fluorescence techniques. Early ensemble luminescence resonance energy transfer experiments did not show a difference in the cleft closure state between both full and partial agonists (9), supporting the hypothesis of a single closed cleft conformation; however, these studies were unable to resolve any difference in the stabilization of the closed cleft state, which would be central to the mechanism of the two-state hypothesis. More recently, single molecule Förster resonance energy transfer (smFRET) has been used to experimentally observe the dynamic changes undergone by the NMDA receptor (2), and specifically by the GluN1 LBD (16, 17). These latter studies provided the first experimental evidence of a partial agonist-dependent change in the conformational equilibrium of the GluN1 LBD; however, the time resolution for these experiments was limited to 10 ms. With the kinetic movements of the GluN1 LBD occurring faster than this resolution (18) and the lack of a clear conformational model, more robust experimental methods were needed to clarify this mechanism of partial agonism.

**FIGURE 1.**
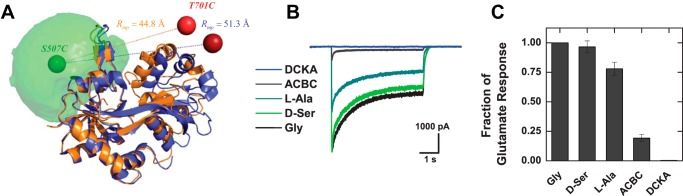
**Experimental smFRET design and construct validation.** *A,* schematic representation of the glycine-bound (PDB code 1PB7, *orange*) and DCKA-bound (PDB code 1PBQ, *blue*) conformations of the GluN1 ligand-binding domain of the NMDA receptor. The AV simulations were calculated to determine the available space that the fluorescent marker will occupy with the donor (Alexa 488) at Ser-507 and acceptor (Alexa 647) at Thr-701. The *green* “AV-cloud” represents all the locations the donor dye can access, and the *green* and *red spheres* represent the mean positions of the dyes for donor and acceptor, respectively, for each structure. The distance between the mean position at each conformation is *R_mp_* = 44.8 and 51.3 Å for glycine and DCKA bound, respectively. Their corresponding expected mean FRET efficiency distances are 〈*R_DA_*〉*_E_* = 48.7 and 54.2 Å. *B* and *C,* whole cell electrophysiological recordings were performed to confirm retained functionality and efficacy of ligands with GluN1 S507C/T701C. *B,* a representative trace shows the reduced efficacy of the two partial agonists, ACBC and l-alanine, relative to the two full agonists, glycine and d-serine, as well as the antagonist DCKA. *C,* group data showing the relative efficacy of each ligand with respect to glycine. Glycine: 100%, d-serine: 94 ± 1%, l-alanine: 84 ± 6%, ACBC: 25 ± 4%, DCKA: 0.3 ± 0.1%.

To probe the conformational landscape of the GluN1 LBD at faster time scales than previously studied, we used smFRET and multiparameter fluorescence detection (MFD) to obtain a complete experimental investigation of the dynamics and conformational equilibrium of the GluN1 LBD. MFD experiments can be used as another method of obtaining smFRET data, but in contrast to obtaining the intensity-based FRET efficiency of individual molecules over a period of seconds, MFD experiments simultaneously measure a number of fluorescence parameters, including intensity, lifetime, and anisotropy of molecules, as they diffuse one at a time through a small confocal volume. The use of time-correlated single photon counting (TCSPC) allows for the exploration of dynamic motions in a broad range of time scales, down to picoseconds (19), making this method particularly well suited for observing the mechanism of partial agonism of the GluN1 LBD. The isolated GluN1 LBD was purified, and site-directed labeling with fluorescent dyes was performed to probe the distance across the LBD cleft (Fig. 1) (16). The results presented here show that the GluN1 LBD exhibits a common closed cleft, active arrangement among a variety of agonists, with partial agonists showing less stability of the closed conformation and more dynamic conversions to the open conformations. Moreover, we find among the FRET states one conformation, which resembles within 2.8 Å the published crystallographic structure for the glycine-bound configuration, and another state that differs only by 1.6 Å from the DCKA-bound structure (13).

## Results

### 

#### 

##### smFRET Experimental Design and Construct Validation

The GluN1 LBD was mutated at Ser-507 and Thr-701 (full-length sequence) on opposite sides of the cleft as has previously been described (16, 17), and then labeled using the FRET pair Alexa 488 and Alexa 647, with an *R*_0_ of 52 Å. Based on crystallographic studies of the glycine-bound (PDB code 1PB7, *orange*) and DCKA-bound (PDB code 1PBQ, *blue*) conformations of the LBD, we performed *in silico* labeling and determined the expected mean FRET efficiency distance 〈*R_DA_*〉*_E_* = 48.7 and 54.2 Å, for both structures, respectively (Fig. 1*A*). With this construct one should be able to observe the clamshell closure due to the binding of different ligands.

To verify that these mutations (GluN1 S507C/T701C) do not abolish the functionality and efficacy of ligands in the full receptor, we obtained whole cell electrophysiological recordings (Fig. 1*B*). Ligand efficacy was determined by normalizing to the maximum amplitude in presence of the full agonist glycine. As expected, d-serine, also a full agonist, has similar efficacy to glycine, followed by l-alanine and ACBC. The last two are considered partial agonists (Fig. 1*C*).

##### Construction of smFRET Histograms

**FIGURE 2.**
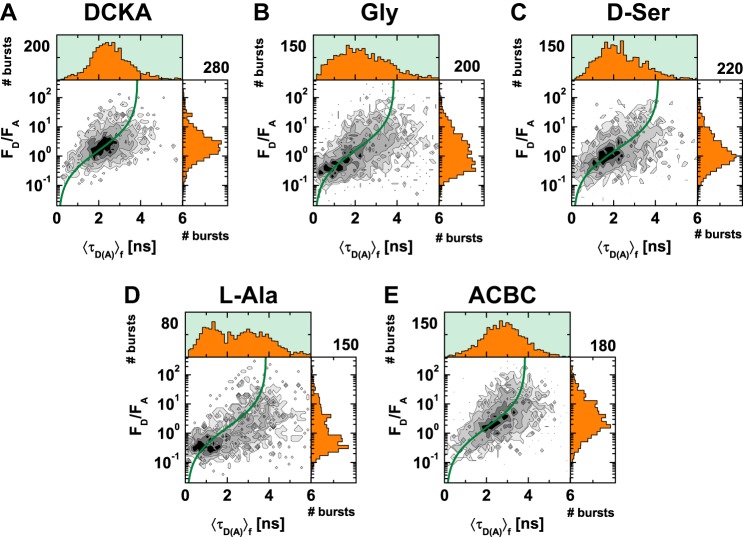
**MFD histograms of labeled GluN1 LBD with multiple ligands.** Two-dimensional single molecule FRET histograms using burst analysis of *F_D_*/*F_A_* distribution *versus* fluorescence averaged lifetime (〈τ*_D_*_(_*_A_*_)_〉*_f_*). The *green line* is the static FRET line, which describes the relationship between *F_D_*/*F_A_* and fluorescence averaged lifetime (〈τ*_D_*_(_*_A_*_)_〉*_f_*). The GluN1 LBD of the NMDA receptor was diluted to picomolar concentrations in the present of various ligands. *A,* 0.1 mm DCKA; *B,* 1 mm glycine; *C,* 1 mm d-serine; *D,* 15 mm l-alanine; and *E,* 10 mm ACBC. The following parameters were used: 〈B_G_〉 Gly = 0.93, 〈B_R_〉 Gly = 0.51, 〈B_G_〉 d-Ser = 0.93, 〈B_R_〉 d-Ser = 0.532, 〈B_G_〉 l-Ala = 0.842, 〈B_R_〉 l-Ala = 0.502, 〈B_G_〉 ACBC = 0.955, 〈B_R_〉 ACBC = 0.518, 〈B_G_〉 DCKA = 0.94 〈B_R_〉 DCKA = 0.522, β = 0.02 (fraction of direct excitation of acceptor with donor excitation laser), α = 0.017, and g_G_/g_R_ = 3.7.

For single molecule experiments, we used pulsed interleaved excitation (PIE) of donor and acceptor fluorophores to excite the doubly labeled LBD. The emitted fluorescence photons were collected to measure various FRET efficiency indicators of single molecules of the LBD when in complex with different ligands (glycine, 1 mm; d-serine, 1 mm; l-alanine, 15 mm; ACBC, 10 mm; or DCKA 100 μm). FRET efficiency was measured simultaneously through both intensity measurements and donor lifetime measurements in the presence of the acceptor (Fig. 2). The resulting single-molecule events or burst histograms are presented in a multidimensional representation, where each event was preselected according to a 1:1 donor to acceptor stoichiometry. The cleaned FRET signal is shown as contours on two-dimensional histograms and as filled histograms over the one-dimensional 〈τ*_D_*_(_*_A_*_)_〉*_f_* and *F_D_*/*F_A_* projections. The green sigmoidal line over the two-dimensional histogram represents the static FRET line (Equation 3, Table 1), which is the theoretical relationship between the two FRET indicators: the donor fluorescence average lifetime 〈τ*_D_*_(_*_A_*_)_〉*_f_* and the ratio of donor-to-acceptor fluorescence (*F_D_*/*F_A_*). Populations that lie on the line indicate FRET states that are “static” (19), *i.e.* populations with dynamic interconversion rates that are slower than the burst duration.

**TABLE 1:** **FRET lines** Equation 3 was used for each different experiment.

Sample	Static FRET line
Gly	(0.7732/0.4240)/((3.8660/((−0.0405 × 〈τ_*D*(*A*)_〉*_f_*^3^) + (0.2914 × 〈τ_*D*(*A*)_〉*_f_*^2^) + 0.4891 × 〈τ_*D*(*A*)_〉*_f_* −0.0422))−1)
d-Ser	(0.8286/0.4290)/((4.1430/((−0.0348 × 〈τ_*D*(*A*)_〉*_f_*^3^) + (0.2676 × 〈τ_*D*(*A*)_〉*_f_*^2^) + 0.4977 × 〈τ_*D*(*A*)_〉*_f_* −0.0443))−1)
l-Ala	(0.8426/0.4130)/((4.2130/((−0.0335 × 〈τ_*D*(*A*)_〉*_f_*^3^) + (0.2622 × 〈τ_*D*(*A*)_〉*_f_*^2^) + 0.4998 × 〈τ_*D*(*A*)_〉*_f_* −0.0448)) − 1)
ACBC	(0.7990/0.3810)/((3.9950/((−0.0377 × 〈τ_*D*(*A*)_〉*_f_*^3^) + (0.2799 × 〈τ_*D*(*A*)_〉*_f_*^2^) + 0.4932 × 〈τ_*D*(*A*)_〉*_f_* − 0.0432))−1)
DCKA	(0.8498/0.3960)/((4.2490/((−0.0329 × 〈τ_*D*(*A*)_〉*_f_*^3^) + (0.2594 × 〈τ_*D*(*A*)_〉*_f_*^2^) + 0.5008 × 〈τ_*D*(*A*)_〉*_f_* −0.0451))−1)

These MFD histograms show clear differences in the conformational landscapes probed by the GluN1 LBD in complex with various ligands. As expected, with the antagonist 5,7-dichlorokynurenic acid (DCKA), mostly medium to low FRET states are explored, with a longer donor fluorescence lifetime and a larger donor-to-acceptor fluorescence ratio (*F_D_*/*F_A_* = 3.3) (Fig. 2*A*). This is consistent with the stabilization of an open cleft conformation. When in complex to the full agonist glycine, the FRET states shift toward higher FRET efficiencies, indicated by lower donor fluorescence lifetimes and smaller donor to acceptor fluorescence ratios (Fig. 2*B*). This is also consistent with the stabilization of the closed cleft conformation. A second full agonist, d-serine, shows a similar trend (Fig. 2*C*), although not as pronounced. To assess the LBD conformational space and dynamics across a variety of activation states we examined two partial agonists (l-alanine and 1-amino-1-cyclobutanecarboxylic acid). Between the two, the more effective partial agonist l-alanine (Fig. 2*D*) resembled more the two full agonists, and the less effective partial agonist 1-amino-1-cyclobutanecarboxylic acid (ACBC, Fig. 2*E*) resembled more the antagonist histogram, similarly to the whole cell recordings (Fig. 1*B*). Of note, the histograms for the two partial agonists seemed to spread across a wider variety of conformational states. These states must be to some extent static because they lie along the green FRET line. It is then evident that none of the ligands trap a single state of the LBD, but rather ligand binding redistributes the population of the conformational states consistent with the mechanism of conformational selection.

##### Probability Distribution Analysis Reveals Three Distinct Conformations

**FIGURE 3.**
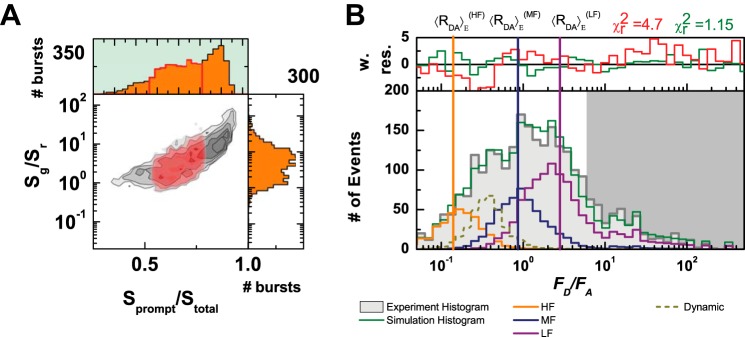
**Photobleaching and description of *F_D_*/*F_A_* histograms as modeled by PDA.** *A,* removing all acceptor photobleaching, due to incorrect signal of prompt channel over all data (0.5< *S*_Prompt_/*S*_Total_ < 0.8). *B,* experimental and PDA-modeled *F_D_*/*F_A_* histogram distributions at Δ*t* = 0.5 ms for the LBD in the presence of glycine. Three limiting states were depicted as Gaussian distributions, each with a different color (high FRET, *orange*, medium FRET, *navy*, and low FRET, *wine*). The mean *F_D_*/*F_A_* value of each distribution is shown as a *vertical line* with the same color code. Each line correlates to the experimentally determined interdye distance per state or 〈*R_DA_*〉*_E_*. One dynamic transition is shown as Gaussian (*dashed dark yellow line*).

To quantitatively analyze the conformational space and dynamic effects induced by ligand binding, we used probability distribution analysis (PDA) (20, 21). We use various models to fit the one-dimensional fluorescence ratio histograms with multiple time window (Δ*t* = 0.5, 2, and 5 ms). In addition, we use PDA to identify the mean FRET efficiency distance (〈*R_D_*_(_*_A_*_)_〉*_E_*) between donor and acceptor for each limiting state. For each conformational state, we use Gaussian distributions that represent the interdye donor-acceptor distance distributions. In PDA analysis, the width (hw_DA_) of each distribution is given by acceptor photophysics (22). To identify the model that best represents the experimental data, we carry a systematic approach of identifying the minimum number of shot-noise limited states (no Gaussian distribution of states). We reached a reasonable convergence with three different FRET states based on visual inspection of the weighted residuals (w. res) and the figure of merit χ^2^. To improve the fit, we added the contribution of the donor-only population due to acceptor bleaching. Although we have burst selection with 1:1 donor-to-acceptor stoichiometry, the presence of Donor only population indicates that a significant fraction of the acceptor is photobleached within the duration of the time window. To identify and remove this artifact further, we use the ratio of the prompt signal corresponding to the TCSPC channels of donor excitation (*S*_prompt_) and total uncorrected signal of donor and acceptor emission over all TCSPC channels (*S*_Total_) (donor and acceptor excitation in PIE experiments) (Fig. 3*A*). It is worth mentioning that the stoichiometry parameter is corrected for quantum yield and detection efficiencies; however, the raw detected signal (*S*) does not require additional corrections. Therefore, this selection serves as an additional identification of events that smear toward the donor only population due to photobleaching. We ruled out the possibility of a very low FRET state due to very long interdye distances because after the *S*_prompt_/*S*_Total_ (Fig. 3*A*) selection there were no left over burst with high enough *F_D_*/*F_A_* ratio and 1:1 stoichiometry.

After identifying the minimum number of FRET-related conformations, we increased the level of complexity in the fitting model. For example, we know that intensity-based FRET parameters are determined by fluctuations on the integrated acquisition time. PDA is particularly susceptible for capturing the blinking behavior of dyes, which produces additional broadening of the distribution beyond the shot noise limit. This behavior has been well characterized (22). It is known that broadening is caused mostly due to acceptor blinking and it follows a monotonic relationship with respect to the interdye separation distance (22). Thus, each FRET-related conformational state will have its own distribution of distances with a particular width (hw_DA_) and mean interdye distance 〈*R_DA_*〉*_E_*. Note that *R_mp_* and 〈*R_DA_*〉*_E_* represent different distances (see accessible volume under “Materials and Methods”). Benchmark studies (23, 24) have shown that 6% of the interdye distance 〈*R_DA_*〉*_E_* is a typical effective width per state. Thus, we fixed the distribution width to 6% of each 〈*R_DA_*〉*_E_*. Broadening beyond this limit would be considered to emerge from dynamic processes.

To exemplify this representation, we show in Fig. 3*B* the experimental and PDA modeled *F_D_*/*F_A_* histogram distributions at Δ*t* = 0.5 ms for the LBD in presence of glycine. Here, we identify three limiting states depicted as Gaussian distributions, each with different color (high FRET *orange*, medium FRET *Navy*, and low FRET *wine*). The mean *F_D_*/*F_A_* value of each distribution is shown as a *vertical line* with the same color code. Each line correlates to the experimentally determined interdye distance per state or 〈*R_DA_*〉*_E_*. In addition to three limiting states, one dynamic transition, also shown as Gaussian (*dark yellow*), is added to statistically improve the fitting quality. For example, in this case χ^2^ decreases from 4.7 to 1.15, when dynamics is included at Δ*t* = 5 ms. Weighted residuals (w. res.) are shown on top layer for visual representation of the goodness of the fit. In a simplified representation it is possible to only show the model distribution as compared with the experimental histogram and the *vertical lines* for representing the mean *F_D_*/*F_A_* value per state. Hereafter, this simplified representation will be used.

##### Time Window Analysis Reveals Submillisecond Dynamics

**FIGURE 4.**
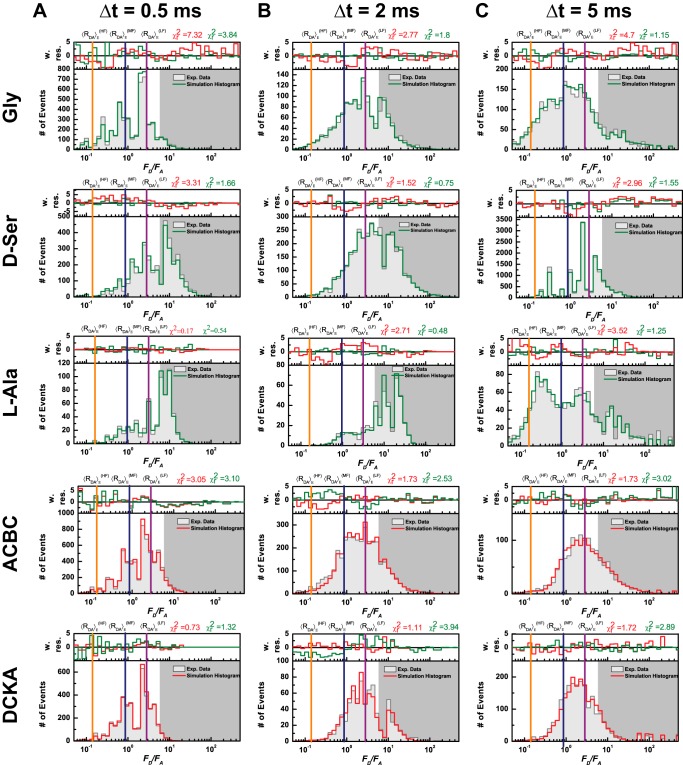
**Time window analysis and PDA comparison *F_D_*/*F_A_* histograms of the LBD with the various ligands.** Time window (Δ*t*) analysis for 0.5, 2, and 5 ms (*A–C*, respectively). The same *F_D_*/*F_A_* correction parameters are used as described in the legend to Fig. 2. The dynamic PDA model consists of three static FRET states (HF, MF, and LF) plus a single two-state kinetic transition between HF and MF. Fractions are renormalized to consider only FRET populations. We observe that glycine has a faster relaxation time compared with l-alanine as glycine equilibrates within the selected time windows. A similar result is seen with d-serine. Splitting of populations occur in the case of l-alanine. Relaxation times are shown in Table 2. *Vertical lines* correspond to the mean FRET efficiency distance of the three limiting states (HF, *orange*; MF, *blue*; and *LF*, *magenta*; Table 2). Donor only or acceptor photobleaching region has a *dark gray background*. Tables 2, 3, 6, and 7 summarize the results from PDA. We determined that glycine has faster relaxation time compared to L-alanine as glycine equilibrates within the selected time windows. A similar result is seen with d-serine. Splitting of populations occur in the case of l-alanine.

To study if there were any dynamic processes involved in the submillisecond to millisecond time scale between states, the experimental *F_D_*/*F_A_* distributions were globally fit using three time windows (Δ*t* = 0.5, 2, and 5 ms). If all states were static within the time window, the static model would roughly fit all time windows equally well and the probability distribution would not change. This was the case for the LBD bound to DCKA and ACBC, suggesting that the states were static within the selected time windows (Fig. 4 for DCKA and ACBC). The figure of merit χ^2^ and the modeled *F_D_*/*F_A_* distribution are shown in *red* when the states are treated as static and it is shown in *green* when the model includes a dynamic transition. If during the selected time window, a molecule switches multiple times between states, the fluorescence bursts of the interconverting molecules will show different degrees of mixing between states; thus changing the probability distribution. This is only true if the dynamic interconversion occurs at time scales that are smaller or comparable to the selected time window. The need for a dynamic state was noticeable for the glycine, d-serine, and l-alanine bound states (Fig. 4), whereas for DCKA and ACBC χ^2^ increases by the addition of the dynamic states.

The larger differences comparing multiple time windows in the distribution for l-alanine are indicative of slower kinetics in the millisecond to submillisecond time scales. It is obvious in this case that there is a split of states with an increase in population of the higher FRET states. Faster kinetics equilibrates the distribution at shorter time windows as seen in the case of glycine because there is no split of states. However, there is evidence of population redistribution toward higher FRET efficiencies or lower *F_D_*/*F_A_* ratios. Therefore, the static model used of three FRET states is no longer valid. To include the dynamic component we tested the addition of a single two-state transition between any FRET states. Remember that the mean *F_D_*/*F_A_* value of each state is shown as a vertical line with the same color code and the relationship to distance can be readily determined (Tables 2 and 3).

**TABLE 2:** **〈*R_DA_*〉*_E_* determined by PDA analysis**

Sample	HF	MF	LF
	Å		
All	33.9	45.8	55.8

**TABLE 3:** ***F_D_*/*F_A_* ratio for each given mean FRET distance**

Sample	HF	MF	LF
Gly	0.14	0.86	2.8
d-Ser	0.15	0.9	2.9
l-Ala	0.16	1.0	3.1
ACBC	0.16	1.0	3.2
DCKA	0.16	1. 0	3.2
*F_D_*/*F_A_* = Φ*_FD_*_(0)_/Φ*_A_* × (*R_DA_*/*R*_0_)^6^ (*R_0_* = 52 Å)			

We observed that the single two-state kinetic state (*HF* ⇌ *MF*) was needed to significantly improve our figure of merit (χ^2^) across time windows for the LBD bound to the full agonists and to the partial agonist l-alanine. In summary, for all cases, we identified three FRET states with the following inter dye distances: the high FRET (HF) (〈*R_DA_*〉*_E_* = 33.9 Å), medium FRET (MF) (<〈*R_DA_*〉*_E_* = 45.8 Å), and low FRET states (LF) (〈*R_DA_*〉*_E_* = 55.8 Å) (Table 2).

**TABLE 4:** **Average steady state anisotropy (*r*_ss_) per burst for the dyes on the ligand binding domain at the conditions** Donor in the absence of acceptor (D only), direct excitation of the acceptor (A), emission of the donor in the presence of acceptor (*D*(*A*)), and sensitized emission of the acceptor by FRET (*A*(*D*)).

*r*_ss_	Gly	d-Ser	l-Ala	ACBC	DCKA
**D only**	0.15	0.13	0.1	0.15	0.11
**A**	0.36	0.06	0.04	0.06	0.07
**D(A)**	0.18	0.18	0.13	0.18	0.15
**A(D)**	0.03	0.04	0.03	0.04	0.05

**FIGURE 5.**
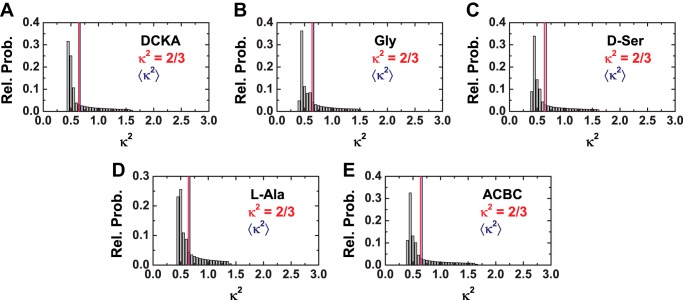
**κ^2^ distribution for LBD bound to: *A,* DCKA; *B,* Gly; *C,*d-Ser; *D,*l-Ala; and *E,* ACBC.** The line for κ^2^ = 2/3 is shown in *red* for each distribution. Mean κ^2^ is shown in *blue*.

These distances were determined with the assumption that κ^2^ = 2/3. To validate this assumption, the κ^2^ distribution and the mean values for these conditions were determined using the wobble in a cone model (Fig. 5). For this, we assume that the residual anisotropies can be approximated in the worst case scenario to the average steady state anisotropy per burst, or 〈*r*_ss_〉 ≅ *r*_∞_, for *D* only (donor), *A* (acceptor), and *A*(*D*) (the sensitized by FRET emission of acceptor) from single molecule experiments (Table 4). We observe that the maximum error introduced by this assumption is 2.5% (Table 5), thus, validating our assumption.

**TABLE 5:** **Mean κ^2^ and estimated error ((*R*_*DA*_^(〈κ2〉)^)/*R*_*DA*_^(κ2=2/3)^%) on distances by using the assumption of κ2 = 2/3**

Sample	〈κ^2^〉	Error
		%
Gly	0.636	2.2
d-Ser	0.631	2.5
l-Ala	0.637	1.8
ACBC	0.634	2.5
DCKA	0.641	2.0

The addition of a two-state kinetic transition (*HF* ⇌ *MF*) occurring in the submillisecond time scales indicate that d-serine exerted the fastest exchange dynamics (*t_R_* = 3.5 μs; Table 6), followed by glycine (*t_R_* = 7.6 μs) and the slowest observed kinetics as expected by the time window analysis was l-alanine with *t_R_* = 50 μs (Table 6).

**TABLE 6:** **Fastest relaxation time observed with PDA**

Sample	*t_R_*
	*ms*
Gly	0.0076
d-Ser	0.0035
l-Ala	0.050

**FIGURE 6.**
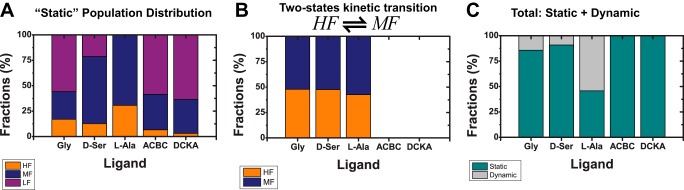
**Redistribution of population fractions.** *A,* the population of the static contribution of FRET states. *B,* a single two-state kinetic state (*HF* ⇌ *MF*) was used to model the additional dynamics oberved. The bar plot shows the distribution of populations of the HF and MF states. The derived distances for high FRET, medium FRET, and low FRET states are 〈*R_DA_*〉*_E_* = 33.9 Å (HF), 〈*R_DA_*〉*_E_* = 45.8 Å (MF), and 〈*R_DA_*〉*_E_* = 55.8 Å (LF) and are shown by *orange*, *blue*, and *purple*, respectively (Table 2). Dynamics fractions were obtained by globally fitting 3 time windows (Δ*t* = 0.5, 2.0, and 5 ms). DCKA and ACBC do not have dynamic contributions. *C,* separation of static and dynamic populations that contribute to the over all scheme. l-Alanine is found more often exchanging at submillisecond time scales.

The dynamic analysis overall suggests that the full agonists glycine and d-serine have rapid dynamic motions specifically associated with the LBD rapidly fluctuating between the MF and HF states, whereas the partial agonist l-alanine has slower dynamics with occasional visits to the HF state. ACBC and DCKA appear static in the millisecond time scale as shown in Fig. 6. ACBC has a slightly higher fraction in the HF state relative to DCKA. Note that static populations represent slow exchange at time scales longer than the burst duration, or trapped states. These data, when correlated to the activation profile, suggest that the visits of the LBD to the HF states are critical for the agonist to activate the channel. This is also consistent with the previously published single channel recordings where it has been shown that partial agonists tend to have longer closed times, which would be consistent with the slower kinetics observed for the partial agonists (2). In addition, when combining the contribution of the static populations and the two-state kinetics between the HF and MF states, we observe that l-alanine is found more often exchanging over these two states more than the two full agonists glycine and d-serine and thus spends less time in the active state. The summary of all populations analysis is presented in Table 7.

**TABLE 7:** **Overall fractions of PDA analysis including the donor only (bleached fraction)**

Sample	HF	MF	LF	Donor only/acceptor bleaching
Gly	59.4	14.4	5.5	20.7
d-Ser	9.7	37	11.1	42.2
l-Ala	13.6	22.8	0.0	63.6
ACBC	3.40	18	30.2	48.4
DCKA	2.0	21.4	40.8	35.8

## Discussion

To investigate the mechanism of partial agonism in the GluN1 subunit of the NMDA receptor, we have measured the cleft opening and closing motion of the LBD of the NMDA receptor in the presence of the full agonists glycine and d-serine, the partial agonists l-alanine and ACBC, and the antagonist DCKA. The presence of the ligands redistributed the state populations, indicative of the conformational selection and preferred state. Even in the presence of ligands the LBD showed dynamic sampling of at least three different FRET conformations that could be separated with our FRET measurements. To quantify the dynamics, we used PDA and time window analysis to provide population analysis and relaxation times of exchange rates between the MF and HF populations. These results show that the LBD when bound to an antagonist spends much of its time in the open-cleft conformation leading to a closed channel. Although there is a significant fraction of the MF state shared in all ligands, it seems that this conformation does not directly lead to activation of the channel. When comparing the measured FRET distance with the expected distances computed from the AV modeling using the crystallographic structure (PDB code 1PB7) we obtain the experimentally determined MF distances as 〈*R_DA_*〉*_E_* = 45.8 Å, whereas the AV expected distance is 〈*R_DA_*〉*_E_* = 48.7 Å. Thus, we can clearly see that the MF population resembles within 2.8 Å the crystallographic structure in the presence of ligand. For the DCKA state (PDB code 1PBQ) we experimentally determined a LF distance 〈*R_DA_*〉*_E_* = 55.8 Å compared with the expected distance of 〈*R_DA_*〉*_E_* =54.2 Å from AV simulations. Again, excellent agreement is found with a 1.6 Å difference.

Moreover, in Fig. 6 one could also observe that, although there are significant changes between various partial agonists and the full agonists, faster kinetics are observed for the full-agonist bound LBD. The relaxation time (*t_R_*) of the glycine-bound LBD is almost an order of magnitude faster than the *t_R_* observed when the LBD is bound to the partial agonist l-alanine. These findings are in good agreement with single-channel recordings that showed longer closed times when bound to partial agonists (18), and faster kinetics is observed for the receptor in the presence of glycine than in the presence of l-alanine. Additionally, the primary three closed states seen in single channel recordings appear to correlate with the three states observed in the smFRET data here, with the HF state being the one more likely leading to channel activation. Thus, the data presented here nicely joins the experimental structural data seen in x-ray crystallography with the experimental functional data of single-channel electrophysiological recordings to create a unified explanation of the mechanism of partial agonism at the GluN1 LBD.

## Experimental Procedures

### 

#### 

##### Electrophysiology

HEK-293T cells were transfected using jetPRIME® Polyplus with GluN1 S507C/T701C, wild-type GluN2A, and enhanced GFP at a microgram ratio of 1:3:1, respectively, with 5 μg of total DNA/10 ml of medium. After a 10–12-h incubation with transfection reagents, cells were plated at low density onto tissue culture dishes. 300 μm dl-APV and 30 μm DCKA were present in the medium during and after transfection. Whole cell patch clamp recordings were performed 24–48 h after transfection using borosilicate glass pipettes with 3–5 megohm resistance, coated with dental wax, fire-polished, and filled with the following solution: 135 mm CsF, 33 mm CsOH, 2 mm MgCl_2_, 1 mm CaCl_2_, 11 mm EGTA, and 10 mm HEPES, pH 7.4. The external solution was: 140 mm NaCl, 2.8 mm KCl, 1 mm CaCl_2_, 10 mm HEPES, pH 7.4. Solutions were locally applied to isolated cells using a stepper motor system (SF-77B; Warner Instruments) with triple barrel tubing. External solution alone was applied as a control, and the cells were then pulsed with glutamate (1 mm) and with a GluN1 ligand for 5 s with a 3-s interval between pulses. The GluN1 ligands tested were glycine, 1 mm; d-serine, 1 mm; l-alanine, 15 mm; ACBC, 10 mm; and DCKA, 100 μm to match the MFD experiments. Cells were held at −60 mV. All recordings were performed using an Axopatch 200B amplifier (Molecular Devices), acquired at 10 kHz using pCLAMP10 software (Molecular Devices) and filtered online at 5 kHz. All experiments were performed at room temperature.

##### GluN1 S1S2 Purification

The original construct for the *Rattus norvegicus* GluN1 S1S2 LBD in pET22b(+) was kindly donated by Eric Gouaux (Oregon Health Science Center, Portland, OR). The codons for serine 115 and threonine 193 of the construct correspond to serine 507 and threonine 701 of full-length GluN1. Both sites were mutated to encode cysteine residues using standard site-directed mutagenesis protocols. This plasmid was then transformed into Origami B (DE3) *Escherichia coli* (Novagen), and cultures of transformed *E. coli* were grown until the culture reached an optical density of 0.8. At this point, protein expression was induced using 0.5 mm isopropyl 1-thio-β-d-galactopyranoside (Fisher), and expression was allowed to proceed at 20 °C for 24 h. Cultures were then harvested, pelleted, and stored at −80 °C until purification.

After thawing, induced *E. coli* pellets were further lysed using a cell disruption vessel (Parr Instruments). Cell debris were pelleted at 185,000 × *g* for 1 h at 4 °C, and the GluN1 S1S2 in the supernatant was loaded onto an immobilized metal affinity chromatography column that had been previously charged with nickel sulfate (HiTrap HP, GE Healthcare) using fast protein liquid chromatography (AKTA, GE Healthcare). Purified GluN1 S1S2 was then eluted using a linear gradient of imidazole (Sigma).

##### FRET Labeling of Purified GluN1 S1S2

Purified GluN1 S1S2 containing cysteines at Ser-115 (Ser-507 full-length sequence) and Thr-193 (Thr-701 full-length sequence) were labeled using Alexa 488 maleimide as the donor and Alexa 647 maleimide as acceptor (Invitrogen). Excess dye was removed by purifying the protein onto a nickel affinity column (nickel-nitrilotriacetic acid-agarose, Qiagen). Imidazole was used for elution and it was removed using a PD-10 desalting column equilibrated with PBS buffer (GE Healthcare). Appropriate concentrations of the specific ligands were then added to the protein for MFD experiments.

##### Accessible Volume (AV) Simulations to Estimate Measured Distance

The accessible volume considers the dyes as hard sphere models connected to the protein via flexible linkers (modeled as a flexible cylindrical pipe) (23–26). The overall dimension (width and length) of the linker is based on their chemical structures. For Alexa 488 the five-carbon linker length was set to 20 Å, the width of the linker is 4.5 Å, and three dye radii 5.0, 4.0, and 1.5 Å. Similarly, for Alexa 647 the dimensions used were: length = 22 Å, width = 4.5 Å and the three dye radii 11.0, 3.0, and 1.5 Å.

To account for dye linker mobility we generated AVs for donor and acceptor dyes attached to the LBD by *in silico* labeling at Ser-507 and Thr-701. For this pair of AVs, we calculated the distance between dye mean positions (*R*_mp_),

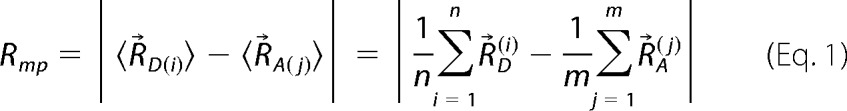
 where *R⃗*_*D*_^(*i*)^ and *R⃗*_*A*_^(*i*)^ are all the possible positions that the donor and acceptor fluorophores can take. However, in intensity based measurements, the mean donor-acceptor distance is determined by the integration time and *R_mp_* cannot be experimentally determined; thus, the effective and experimentally determined distance becomes,

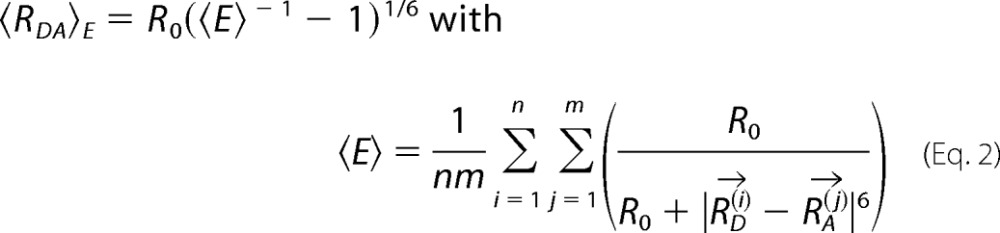
 the relationship between *R_mp_* and 〈*R_DA_*〉*_E_* can be derived empirically following a third order polynomial from many different simulations.

##### MFD for smFRET Experiments

MFD for confocal smFRET studies of single molecules was done using PIE (27) with two diode lasers (model LDH-D-C-485 at 485 nm and laser LDH-D-C-640 at 640 nm; PicoQuant, Germany) operating at 40 MHz with 25-ns interleaved time. The power at objective was set for 120 microwatts for the 485-nm laser line and 39 microwatts for the 640 nm excitation. Freely diffusing doubly labeled LBD molecules are excited as they pass through the focal point of a ×60, 1.2 NA collar (0.17) corrected Olympus objective. The emitted fluorescence signal was collected through the same objective and spatially filtered using a 70-μm pinhole to define an effective confocal detection volume. The emitted fluorescence was divided into parallel and perpendicular polarization components at two different spectral windows (“green” and “red”) through band pass filters, ET525/50 and ET720/150, for green and red, respectively (Chroma Technology Co.). In total, four photon detectors are used: two for green (PMA hybrid model 40 PicoQuant) and two for red channels (PMA hybrid model 50, PicoQuant). A TCSPC module (HydraHarp 400, PicoQuant) with time-tagged time-resolved mode and 4 synchronized input channels were used for data registration.

For smFRET measurements donor-acceptor (DA)-labeled LBD samples were diluted to a picomolar concentration in PBS buffer (50 mm sodium phosphate, pH 7.5, 150 mm NaCl), which had been charcoal filtered to remove residual impurities. At picomolar concentrations we assure that we observe ∼1 molecule/s. To prevent adsorption artifacts, NUNC chambers (Lab-Tek, Thermo Scientific, Germany) were pre-coated with a solution of 0.01% Tween 20 (Thermo Scientific) in water for 30 min and then rinsed with ddH_2_O. Collection time varied from several minutes up to 2 h. Standard controls consisted of measuring water to determine the instrument response function, buffer for background subtraction and the nanomolar concentration of green and red standard dyes (Rhodamine 110, Rhodamine 101, and Alexa 647) in water solutions for calibration of green and red channels, respectively. To calibrate the detection efficiencies we used a mixture solution of double labeled DNA oligonucleotides with known distance separation between donor and acceptor dyes. Ligands used were glycine, 1 mm; d-serine, 1 mm; l-alanine, 15 mm; ACBC, 10 mm; or DCKA 100 μm.

##### MFD Histograms and FRET Lines

Bursts were selected by 2σ criteria out of the mean background value with cut-off times that vary from sample to sample with a minimum of 60 photons for each burst (28, 29). Each burst was then processed and fitted using a maximum likelihood algorithm and previously developed programs (LabVIEW, National Instruments Co.) (30). Bursts were selected according to the following rules: the difference in burst duration on green channels given donor excitation (*T*_GX_) and burst duration on red channels given direct acceptor excitation (*T*_RR_) was −1.5 ms < *T*_GX_ − *T*_RR_ < 1.5 ms; and bursts satisfy the FRET stoichiometry (*S*_PIE_) parameter of 0.13 < *S*_PIE_ < 0.6, which selects for bursts with both fluorophores present. Fluorescent bursts were plotted in two-dimensional histograms (Origin 8.6, OriginLab Co.).

The relationship between the ratio of the donor fluorescence over the acceptor fluorescence *F_D_*/*F_A_* and the fluorescence-weighted lifetime obtained in burst analysis 〈τ*_D_*_(_*_A_*_)_〉*_f_* depends on specific experimental parameters such as fluorescence quantum yields of the dyes (Φ_*FD*(0)_ and Φ*_FA_* for donor and acceptor, respectively), background (〈B_G_〉 and 〈B_R_〉 for green and red channels), detection efficiencies (g_G_ and g_R_ for green and red, respectively), and cross-talk (α). The parametric line that relates two FRET indicators (*F_D_*/*F_A_* and 〈τ*_D_*_(_*_A_*_)_〉*_f_*) was introduced by Seidel's group and is defined as,

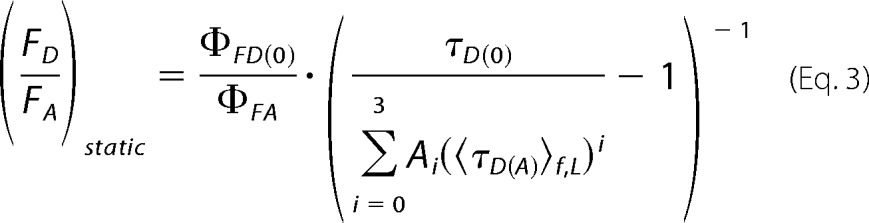
 where *A_i_* are the coefficients of an empirical polynomial function that takes into account the intrinsic linker dynamics of the dyes. FRET lines are used to identify static or slowly exchanged limiting populations.

##### Quantum Yields

The donor and acceptor quantum yields were corrected due to the presence of different ligands and need to be corrected accordingly. We assumed that only dynamic quenching takes place and that Φ*_FD_*_(0)_, Φ*_FA_* are proportional to the species-averaged fluorescence lifetime 〈τ*_D_*_(_*_A_*_)_〉*_x_* of donor or acceptor, respectively. As reference samples we used Alexa 488-labeled DNA 〈τ*_D_*_(0)_〉*_x_* = 4.0 ns, Φ*_FD_*_(0)_ = 0.8 and for the acceptor we used Cy5-labeled DNA with 〈τ*_A_*〉*_x_* = 1.17 ns and Φ*_FA_* = 0.32 (31). The obtained donor and acceptor quantum yields are presented in Table 8. This FRET pair has a reduced Förster distance of 52 Å where we assumed isotropic reorientation of the dyes using κ^2^ = 2/3 due to the long linkers.

**TABLE 8:** **Quantum yields were estimated as described under “Experimental Procedures”**

Sample	Φ*_FD_*_(0)_	Φ*_FA_*
Gly	0.773	0.42
d-Ser	0.828	0.43
l-Ala	0.843	0.41
ACBC	0.799	0.38
DCKA	0.850	0.40

##### κ^2^ = 2/3 Assumption, 〈κ^2^〉, and κ^2^ Distributions

Experimentally, one can test whether assuming κ^2^ = 2/3 is justifiable or not. Considering that fluorophores follow the “wobble in a cone” model (32), it is possible to calculate a distribution of all possible values of κ^2^. For that, we determined the residual anisotropies (*r*_∞_) (D only, donor; A, acceptor, and A(D), the sensitized by FRET emission of acceptor) from single molecule experiments. We consider the extreme limit when 〈*r*_ss_〉 ≅ *r*_∞_. Then, all κ^2^ values will follow (23),

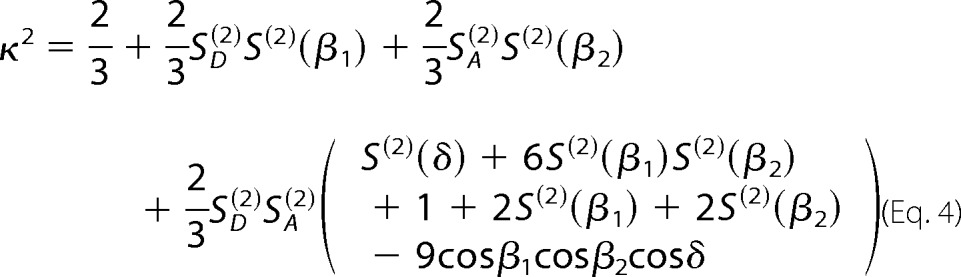
 where, β_1_ and β_2_ are the angles between the symmetry axes of the dyes rotations, and δ is the angle between the symmetry. The necessary second-rank order parameters *S*^(2)^ are defined by,

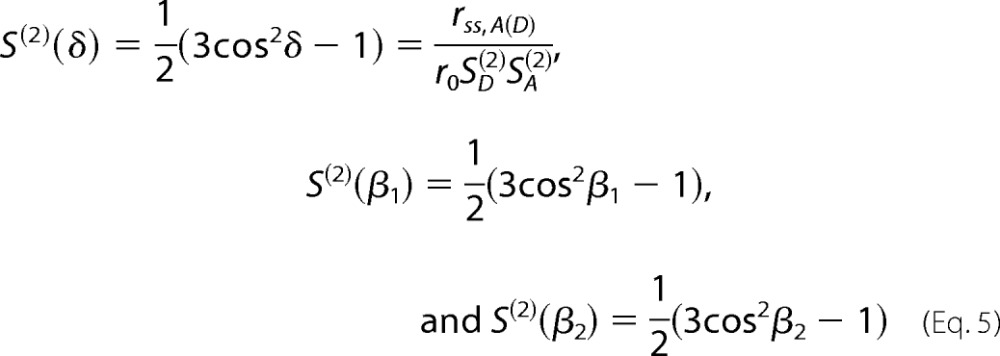
 where *r*_0_ is the fundamental anisotropy of the dyes whose values were 0.38 and 0.39 for the donor and acceptor fluorophores, respectively. The dye motions are characterized by the second-rank order parameters *S*_*D*_^(2)^ and *S*_*A*_^(2)^ by Equation 6.

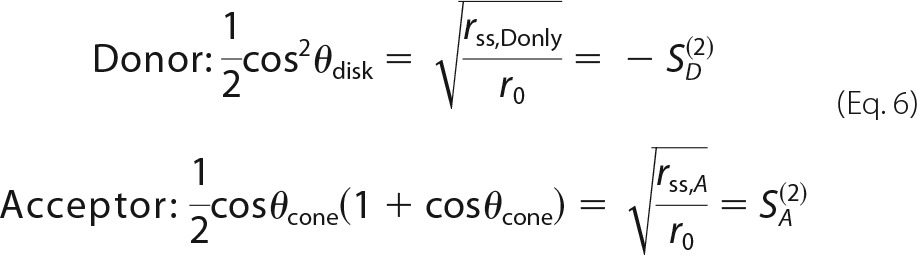
 From all possible orientations and combinations a κ^2^- distribution and its corresponding arithmetic mean (〈κ^2^〉) can be determined and compared with the assumed to κ^2^ = 2/3.

##### PDA Analysis

To model the shape of the *F_D_*/*F_A_* distributions we use probability distribution analysis or PDA (20, 33). In short, the measured fluorescence signal (*S*), consisting of fluorescence (*F*), and background (*B*) photons are expressed in photon count numbers per time window (Δ*t*) of a fixed length. In multiparameter fluorescence detection the signal is split into two spectral windows termed “green” and “red” each with two polarization components (parallel “‖” and perpendicular “⊥”). The probability of observing a certain combination of photon counts in two detection channels 1 and 2 (*e.g.* 1 = green and 2 = red) and measured by two or more single-photon counting detectors, *P*(S_1_,S_2_), is given by a product of independent probabilities,

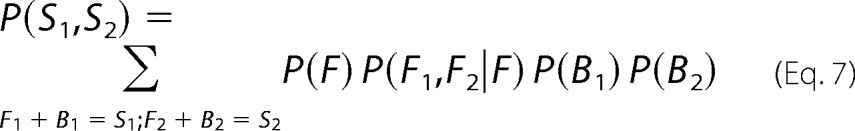

*P*(*F)* describes the fluorescence intensity distribution, *i.e*. the probability of observing exactly *F* fluorescence photons per time window (Δ*t*). *P*(*B*_1_) and *P*(*B*_2_) represent the background intensity distributions. *P*(*F*_1_,*F*_2_|*F*) is the conditional probability of observing a particular combination of *F*_1_ and *F*_2_, provided the total number of fluorescence photons is *F*. This can be expressed as,

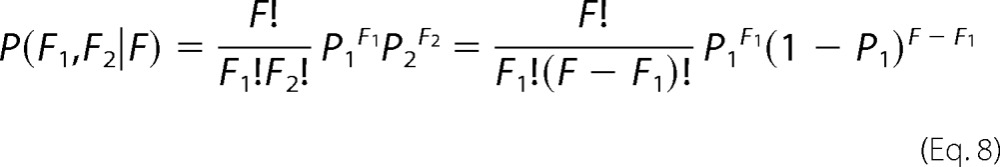
 where *p*_1_ stands for the probability of a detected photon to be registered by the first detector (*e.g.* green in a FRET experiment). For smFRET, *p*_1_ is unambiguously related to the FRET efficiency *E* according to,

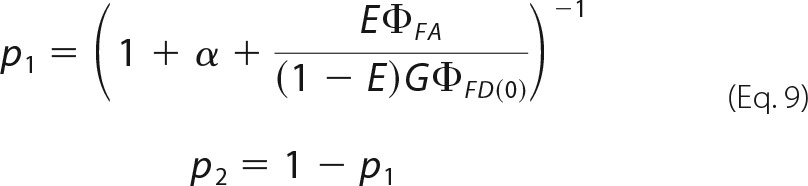
 here, *G* stands for the ratio of the detection efficiencies in the spectral windows (*G* = g_G_/g_R_). The quantum yields (Φ*_FD_*_(0)_ and Φ*_FA_*) were previously defined, and α is the spectral cross-talk.

The distribution *P*(*F*) in Equation 7 is not directly measurable; instead, the total signal intensity distribution *P*(*S*) is measured, which is given by,

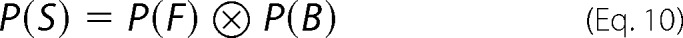
 where *P*(*B*) is the distribution probability of background counts. Details on the deconvolution procedure are described elsewhere (20). Finally, Equation 9 can be extended for multiple species with the brightness correction used in this work (34). Each species distribution has a half-width (hw_DA_), which depends mostly in shot noise and photophysical properties of the acceptor fluorophore, and it was fixed to 6% of the 〈*R_DA_*〉*_E_*.

## Author Contributions

D. M. D. grew, purified, and labeled the GluN1 LBD. S. R. A. performed the MFD experiments and analysis. S. A. S. performed the electrophysiology experiments and analysis. V. J. conceived the experiments and supervised the protein biochemistry. H. S. supervised the MFD experiments and analysis. All authors participated in the design and direction of the project, and all authors contributed to the writing of the manuscript.

## Footnotes

4 The abbreviations used are:

LBD: ligand-binding domain
smFRET: single molecule Förster resonance energy transfer
MFD: multiparameter fluorescence detection
ACBC: 1-amino-1-cyclobutanecarboxylic acid
DCKA: 5,7-dichlorokynurenic acid
PDA: probability distribution analysis
TCSPC: time-correlated single photon counting
PIE: pulsed interleaved excitation
AV: accessible volume
PDB: Protein Data Bank
LF: low FRET
MF: medium FRET
HF: high FRET.
